# Nanobubbles Water Curcumin Extract Reduces Injury Risks on Drop Jumps in Women: A Pilot Study

**DOI:** 10.1155/2019/8647587

**Published:** 2019-04-01

**Authors:** I-Lin Wang, Chien-Yu Hsiao, Yu-Heng Li, Fan-Bo Meng, Chi-Chang Huang, Yi-Ming Chen

**Affiliations:** ^1^Health Technology College, Jilin Sport University, Changchun 130022, Jilin, China; ^2^Department of Nutrition and Health Sciences, Chang Gung University of Science and Technology, Taoyuan 33301, Taiwan; ^3^Research Center for Industry of Human Ecology and Research Center for Chinese Herbal Medicine, College of Human Ecology, Chang Gung University of Science and Technology, Taoyuan 33301, Taiwan; ^4^Aesthetic Medical Center, Department of Dermatology, Chang Gung Memorial Hospital, Taoyuan 33301, Taiwan; ^5^Cardiovascular Department, China‐Japan Union Hospital, Jilin University, Changchun 130033, China; ^6^Graduate Institute of Sports Science, National Taiwan Sport University, Taoyuan 33301, Taiwan

## Abstract

**Purpose:**

To verify the beneficial effects of Nanobubbles water curcumin extract (NCE) supplementation on health promotion and to demonstrate the application of NCE in reducing the risk of musculoskeletal injury.

**Methods:**

In the current study, 12 females were randomly assigned to NCE (15g/day) and maltodextrin groups. Performance and related body composition were evaluated at 2 time points—presupplementation (pre-) and after 4 weeks of postsupplementation (post-). The posttest consists of a set of biochemical parameters for antifatigue activity and injury status evaluation.

**Results:**

NCE group exhibited significantly lower levels of alanine aminotransferase (ALT), alkaline phosphatase (ALP), triglycerides (TG), and higher high-density lipoprotein (HDL) after a 4-week supplementation, compared with the placebo group. After a 15-minute session on the spinning bike, serum lactate and ammonia levels were decreased and glucose was economized in the NEC group. 4-week-NCE supplementation was also able to reduce the peak vertical ground reaction force (PVGRF) during drop jump. Therefore, the risk of musculoskeletal system in lower extremity could be reduced.

**Conclusion:**

We demonstrate that 4-week-NCE supplementation can also be used in explosiveness exercise for better physiological adaptation. Thus, NCE has potential for use with nutrient supplements toward a variety of benefits for athletics.

## 1. Introduction

The nanobubbles (NBs) or microbubbles (MBs) technology in combination with the coagulation/flocculation process has been used effectively to improve the efficiency of drug delivery [1]. Nanobubbles is produced by collapsing microbubble (less or equal to 50 *μ*m in diameter) in the electrolyte under ultra-high temperature and pressure to form gas nuclei <100 nm in diameter [2]. Several studies have shown the use of these NBs water in medical applications: NB water increases blood–brain barrier permeability [3], is used as an adjunct to periodontal treatment due to its potent antimicrobial effect [2], and has been reported to help in the prevention of kidney stones [4].

The main constituent of turmeric (Curcuma longa), curcumin, has been extensively used as a spice, food preservative, and coloring material in China, India, and South East Asia [5]. Also used in traditional medicine, curcumin has a broad range of exercise-related bioactivities including being used as an ergogenic aid for exercise performance [6], reducing endurance exercise-induced inflammation [7], and mitigating the negative effects associated with eccentric exercise-induced muscle damage [8, 9]. In this study, we attempt to use the unique NBs technology in the development of a nanobubble water curcumin extract (NCE) for use as an ergogenic aid.

Certain substances or drugs are termed ergogenic; i.e., they are able to enhance performance by improving exercise energy efficiency, strength, condition, and endurance [10]. Ergogenic aids have been used for centuries, although there has been growing scientific evidence and support for their effects. Athletes are increasingly turning to supplement to improve exercise performance. Their most common motivation is to improve attention, reduce reaction time and delay fatigue, reduce sensitivity to pain, and increase strength, training endurance, and alertness [11] The performance of sports is broadly affected by neuromuscular functions (muscle strength, explosiveness, coordination, energy output (anaerobic, aerobic capacity), and joint mobility (muscle ductility) [12]. The use of these supplements is to be able to enhance both muscle function and motor performance. In order to achieve the highest performance in competitions, athletes need to shorten the compensatory period during long-term continuous exercise so as to accelerate or promote the body's recovery process. Ergogenic substances are able to reduce energy consumption, restore the internal dynamic balance environment, restore body mechanisms for nutrient regeneration, and optimize functional parameters to improve performance [13].

In our previous study of animal study, we demonstrated that curcumin could significantly increase exercise performance and mitigate the increase of physiological biomarkers induced by intensive aerobic training [6]. In the current clinical trial, we examine the antifatigue activity and beneficial effects of nanobubbles water applied on curcumin extract to enhance explosiveness without a training program. NCE supplementation may be helpful for athletes performing explosiveness-type exercises, as well as for overall physiological protective effects.

## 2. Materials and Methods

### 2.1. Preparation of Nanobubble Water Curcumin Extract (NCE)

Nanobubbles (NBs) were generated by using a controllable platform of instant high pressure based on high speed grinding and cutting of water molecules in the presence of a magnet at 3000-4000 E. The NCE extraction process and formula (Leading curcumin®) was provided by Leading Auto. Bio Co. Ltd. (Hsinchu, Taiwan). Curcumin powder was soaked in NBs for 3-5 min until the NBs impacted all curcumin particles. After the NBs process, the sample was dried and stored at room temperature in a dark and dry cabinet. NCE was freshly prepared before each daily administration. The main constituents of curcumin, bisdemethoxycurcumin, and demethoxycurcumin were determined by high‐performance liquid chromatography (HPLC) [14, 15]. The nutritional content and total amount of curcumin in NCE were analyzed by SGS Taiwan, Ltd. (New Taipei City, Taiwan).

### 2.2. Experimental Design

This single center, randomized, placebo-controlled double-blind, parallel group trial was approved by the Joint Institutional Review Board of Jilin Sport University (Changchun, China; JLSU-IRB no. 2018001). Subjects used in this study were volunteers from Jilin Sport University (JLSU). The subjects gave informed consent for inclusion before enrolment into the study which was conducted in accordance with the Declaration of Helsinki. After enrolment, subjects were randomly allocated to 2 groups: nanobubbles water curcumin extract (NCE) or placebo (maltodextrin). Each group (n=6) consumed NCE (containing 230.9 mg curcumin) or placebo at 15 g/day daily before breakfast for 4 consecutive weeks. Both NCE and placebo were isocaloric at 43 kcal/day. Subjects attended 2 study visits on Day 0 (pretest) and Day 28 (posttest). The posttest visit was arranged after the last supplementation. Body composition and effects on exercise performance were measured at these visits [16].

### 2.3. Subjects

Twelve female Jilin Sport University students volunteered to participate in this study. All subjects have no regular exercise habit and individuals were excluded from the study if they had any known metabolic disorders, heart/cardiopulmonary diseases, diabetes, thyroid disease, hypogonadism, hepatorenal disease, musculoskeletal disorders, neuromuscular/neurological diseases, autoimmune diseases, cancer, peptic ulcers, or anemia. Subjects were asked to refrain from any aerobic or anaerobic exercise training while keeping their normal dietary pattern and daily caloric intake under control during the experimental period. Nutritional supplements including certain types of protein, antioxidants, creatine, and steroid supplementation were disallowed. Subjects were randomly assigned to the placebo or NCE group and the basic characteristics of age (21.2 ± 1.1 years), height (166.3 ± 4.5 vs. 165.2 ± 5.0 cm), and weight (57.1 ± 5.1 vs. 56.5 ± 7.6 kg) did not significantly differ before and after the study.

### 2.4. Drop Jumps with Kinetic and Kinematic Analysis

**Figure 1:**
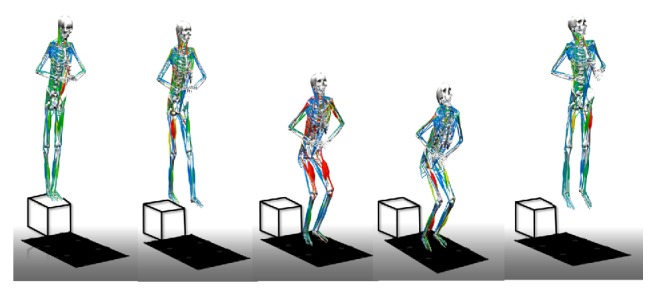
Schematic of the study design on drop jumps (DJs). Subjects had their hands on their waist and performed the DJs by stepping off a raised platform with their dominant leg sticking out from the platform. The body leans forward naturally to drop onto 2 force plates with each foot on separate plates. They were instructed to jump up from the ground with maximum effort after the first landing.

All subjects had to first complete the familiarization day to familiarize themselves with the test protocol and specific jumping techniques. The subjects were attired in shorts, sports bras, and indoor sneakers. Prior to the test protocol, the subjects had to warm up by stretching their high knees and lower jaw, performing gait swings and running for 20 minutes. After the warm-ups, there was a 3-minute rest before the subjects had to carry out 3 repeated counter-movement jumps (CMJs) tests [17]. The subject stood on the force plate and immediately leaped up vertically with maximum force as quickly as possible. There is a 60-second static recovery between each iteration. The highest jump height is defined as 100% high (H). The drop jumps high (DJH) 70%, DJH 100%, and DJH 130% tests were performed three times consecutively with a minute static rest within each DJH repetition and three minutes of interval rest (whereby the subject sat on a chair) between the DJHs. Each subject repeated this entire CMJs test three times. The schematic of DJs is as shown in Figure 1. Subjects are at the top of a raised platform box and step off the platform using their leg of choice. Subjects need to land on their feet at the same time, touch the ground as little as possible, and bounce as quickly as possible to jump to the maximum height. The results are expressed as the average of three performances. The kinetic and kinematic data were collected using the BTS motion capture and analog data acquisition system (Bioengineering, Milano, Italy). Ten infrared Qualisys motion capture cameras (SMART-DX400, BTS Bioengineering, Milano, Italy) at a 200-Hz sampling rate and 4 force platforms (BTS P6000, BTS Bioengineering, Milano, Italy) at a 400-Hz sampling rate were used. Cameras and force platforms were synchronized using a BTS A/D board. A modified Helen Hayes marker set was used to identify the 7-segment rigid link model of the lower extremities.

### 2.5. Acute Spinning Bike Challenge Followed by Biochemical Analyses

Subjects were pretreated with placebo or NCE for 4 weeks before being given the postexercise performance test, given 1 h after the last consumption of study product. Twelve subjects performed a 15-minute test on a spinning bike (Keiser M3i Indoor Cycle, Keiser®, Fresno, California, USA). All subjects wore a Polar T31 heart rate sensor (Polar Electro, Kempele, Finland) while being tested on the spinning bike. The heart rate was under the 60-65% maximum heart rate. The intensity of spinning bike was 85.5 ± 1.7 RPM, 26.9 ± 1.6 Watts, 6.4 ± 0.1 km. After the 15min spinning bike challenge, the biochemical levels of lactate, ammonia (NH_3_), creatine kinase (CK), and glucose were assessed using the Selectra Pro XL analyzer (Vita lab, Netherlands).

### 2.6. Clinical Biochemistry

At the end of experiment, blood samples were taken after subjects had fasted for at least 8 hours. Biochemical levels of aspartate aminotransferase (AST), alanine aminotransferase (ALT), alkaline phosphatase (ALP), lactic dehydrogenase (LDH), creatine kinase (CK), albumin, total protein (TP), blood urea nitrogen (BUN), uric acid (UA), glucose, total cholesterol (TC), triglycerides (TG), high-density lipoprotein (HDL), and low-density lipoprotein (LDL) were assessed using a Beckman Coulter AU5800 autoanalyzer (Beckman Coulter Inc., Brea CA, USA).

### 2.7. Anthropometric Measurements

In the beginning of experiment and at the end of experiment, all subjects arrived at the laboratory in the morning for anthropometric measurements including body height (cm), body weight (kg), body mass index (BMI; kg/m^2^), basal metabolic rates (BMR), free fat mass (FFM, kg), body fat percentage (%), water content percentage (%), and bone salts (kg). Standing body height without shoes or socks was measured to the nearest 0.1 cm with a height meter mounted on a wall. BW, FFM, body fat, water content, and bone salts were measured by a bioelectrical impedance instrument (CH18, HUAWEI Technologies Co., Ltd., Shenzhen, China) using standard methods to assess the body composition.

### 2.8. Statistical Analysis

Statistical analyses were performed using SPSS version 18.0 software (SPSS, Chicago, IL, USA). Data is expressed as the mean ± SEM. A mixed design two-way analysis of variance (ANOVA) (supplementation x time) was used to compare the variables of biochemistry, body compositions, and exercise performance. A* p*-value < 0.05 was considered statistically significant. A post-hoc independent-repeated t-test was used when the main effect or interaction effect was significant. Kinematic and GRF data were filtered using a fourth-order, zero-lag, low-pass Butterworth filter with cutoff frequencies of 10 and 50 Hz, respectively. Initial contact was identified with a vertical GRF threshold of 20 N. The jumping height (H) was calculated using the formula: H = gT^2^/8. The reactive strength index (RSI) was calculated using the following formula: RSI = H/ground contact time. The ground contact time was calculated as the time from the foot leaving the ground to the time of the foot contacting the ground. The peak vertical ground reaction force (PVGRF) was defined as the maximum PVGRF during the first landing phase and the PVGRF was normalized by body weight (BW).

## 3. Results

### 3.1. The Nutritional Content of Nanobubbles Curcumin Extract (NCE)

Total calories of NCE were 43 kcal/15g. The 15g NCE had protein, fat, saturated fat, transfat, carbohydrate, sugar, fiber, and sodium were 1.15 g, 0.21g, 0.06g, 0 g, 10.8 g, 0.22g, 3.46g, and 176.1 mg. The contents of curcumin, bisdemethoxycurcumin, and demethoxycurcumin were 230.9 mg, 56.2 mg, and 75.6 mg in 15g NCE.

### 3.2. Effects of Supplements on Biochemical Variables

Biochemical analyses at the indicated points of the experiment can provide clinical information about the physiological adaptation status of subject. To this end, we categorized different parameters according to physiological functions (Table 1). At the posttest assessment, both hepatic indexes ALT and ALP were significantly decreased in the NCE vs placebo groups by 35.66% (*p*=0.0002) and 28.65% (*p*=0.0013), respectively. For lipid-related parameters, TG showed a significant decrease of 31.96% (*p*=0.0006) in the NCE group compared with the placebo group. The NCE group had significantly increased HDL by 1.17-fold (*p*=0.0017) compared to the placebo group after the 4-week NCE supplementation. The remaining biochemical parameters including AST, LDH, CK, albumin, TP, BUN UA, glucose, TC, and LDL showed no significant difference in the 2 groups.

### 3.3. Effects of Supplementation on Body Composition Profiles

Table 2 shows the body weight (BW), body mass index (BMI), free fat mass (FFM), fat mass, water content, and bone salts content for the NCE and placebo groups as assessed before and after test. The mean height of the subjects in the placebo and NCE group was 166.3 ± 4.5 cm and 165.2 ± 5.0 cm, respectively. At the pretest assessment, the body composition parameters of the subjects in the NCE group were not significant different from the placebo group for BW (*p*=0.8788), BMI (*p*=0.9836), FFM (*p*=0.5370), fat mass (*p*=0.7762), water content (*p*=0.5350), and bone salts (*p*=0.5294). At the end of the 4-week supplementation, both NCE and placebo groups were assessed again for the same parameters. The posttest BW (*p*=0.9170), BMI (*p*=0.9615), FFM (*p*=0.5755), fat mass (*p*=0.7327), water content (*p*=0.8376), and bone salts (*p*=0.7264) were again no different in the 2 groups. The data also showed no significant difference in main (supplementation, times) or interaction effects (supplementation x times). The results suggest that NCE did not significantly affect body composition.

### 3.4. Effects of Supplementation on Exercise Performance

Drop jumps (DJs) from different heights have been commonly used in plyometric training programs. Our results show that NCE groups cannot increase the jumps high (Table 3). Ground reaction force is an important factor affecting ground load. If the landing period results in a large ground reaction force, it will increase the risk of lower extremity and knee injuries [18, 19]. For the muscle strength indexes (contact time), the statistical analysis demonstrated significant supplementation main effects (*p*=0.0379), time main effects (*p*=0.1238), and interaction effects (*p*=0.4630). The posttest analysis showed that the contact time at drop jumps high 100% was significantly increased (*p*=0.0487) by NCE supplementation. The increase in contact times was due to the enhancement of muscle strength by NCE supplementation. The knee injury index (peak vertical ground reaction force, PVGRF) in the posttest DJH 70%. was significantly decreased by 15.38% (*p*=0.0467) in the NCE group compared with placebo group. Taken together, our results suggest that 4-week NCE supplementation increased drop jump's ground contact times and decreased ground reaction force to reduce the risk of injury on drop jumps.

### 3.5. Effects of Supplementation on Serum Lactate, Ammonia, Glucose, and CK Levels after Spinning Bike Challenge

**Figure 2:**
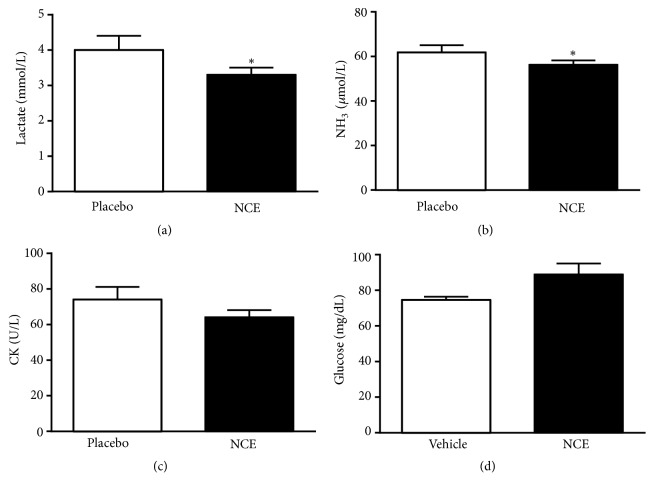
Effect of 4-week-NCE supplementation on serum levels of (a) lactate, (b) ammonia, (c) creatine kinase (CK), and (d) glucose after an acute exercise challenge. Subjects consumed either NCE or placebo for 4 weeks and, one hour following the last administration, a 15-minute spinning bike test was carried out. Data are expressed as mean ± SEM with six subjects in each group. Significance (*∗*) was set at p < 0.05 by a One-Way ANOVA.

Muscle fatigue after exercise can be evaluated by biochemical indicators including lactate, ammonia, glucose and CK levels [20]. Lactate is an oxidizable substrate in skeletal muscle and a precursor to gluconeogenesis in muscles or liver after exercise [21]. In the present study, lactate levels in the placebo and NCE groups were 4.02 ± 0.43 and 3.27 ± 0.18 mmol/L, respectively, with the NCE treatment leading to significantly lower levels (18.67%,* p* = 0.0057) (Figure 2(a)) than the placebo treatments. Peripheral and central fatigue levels are related to increased ammonia level during exercise. Serum ammonia levels in the NCE group were significantly lower (9.02%,* p* = 0.0048) than the placebo group at 61.9 ± 3.2 vs 56.3 ± 1.9 *μ*mol/L (Figure 2(b)). Once muscle glycogen is depleted or near depletion, fatigue sets in and exercise capacity is compromised [22]. Therefore, blood glucose level is an important index for performance maintenance during exercise [23]. Serum CK is an important clinical biomarker for muscle damage such as muscular dystrophy, severe muscle breakdown, myocardial infarction, autoimmune myositis, and acute renal failure. CK activities in the placebo and NCE groups were not significantly different at 74 ± 7 and 64 ± 4 U/L, respectively (Figure 2(c)). Serum glucose levels in the placebo and NCE groups were 74.5 ± 1.9 and 88.9 ± 6.1 mg/dL, respectively, with the NCE group higher by 1.19-fold (*p* = 0.0474) than the placebo group (Figure 2(d)). Our results indicate that NCE supplementation is able to ameliorate skeletal muscle injury induced by acute exercise challenge. Compared to the placebo, NCE supplementation was able to reduce serum lactate, ammonia, and CK levels after the spinning bike challenge, possibly acting as an ergogenic supplement for fatigue recovery and reducing muscle damage.

## 4. Discussion

In the current clinical study, we found that NCE could significantly mitigate ALT, ALP, and TG levels. In addition, NCE also increases serum HDL content (Table 1). NCE supplementation demonstrated functional activities in terms of physiological protection and recovery promotion. NCE supplementation had no adverse effect on body composition with 4-week supplementation (Table 2) and could improve fatigue after a 15-minute spinning bike test as assessed by the biochemical markers. The combination of physiological protection and antifatigue activity can contribute to significant improvement in exercise performance.

In the presence of nanobubbles, the porous nature of the material increases drastically and causes changes in the original properties of the material. As the density of the material changes, its fluidity and other properties are changed [24]. In recent years, nanobubbles (NBs) are spontaneously generated by using a controllable platform of superfast microvortices, based on turbulent jet flows in the presence of graphene oxide sheets [25]. This process is costly and space inefficient; we were able to overcome these problems to develop a rapid and continuous process for application in the nanobubble extraction technology.

Our data suggests that NCE can be applied to a living body for use in fatigue recovery. During high jumps or exhaustive exercises, membrane permeability is altered and large amounts of enzymes such as CK, LDH, AST, and ALT can leak out due to muscle damage [26]. Previous studies have demonstrated the functional bioactivities of curcumin in preventing joint inflammation [27], attenuating exercise-induced oxidative stress by increasing blood antioxidant capacity [28, 29] and reducing delayed onset muscle soreness [30]. In the current study, we also found that 4 weeks of NCE supplementation were able to ameliorate the increase in indexes related to muscle damage. A study of lipids and lipid proteins in aerobic athletes showed characteristics of low TG, low LDL, and high HDL-cholesterol compared to those with a sedentary lifestyle [31]. In the current study, NCE supplementation also significantly decreased TG and increased HDL levels compared to the placebo group, even without exercise training.

Physiological fatigue resulting from inadequate rest, physical loading or mental strain/pressure and is further classified as central or peripheral fatigue [32]. Peripheral fatigue can be assessed by important serum indicators such as lactate, ammonia, glucose, CK, BUN, ALT, and ALT that are related to exercise fatigue or injury [33]. We found that after 4 weeks of supplementation, the NCE group exhibited significantly lower lactate, ammonia, and economic glucose levels after the 15-minute spinning bike challenge compared with the placebo group, indicating that the NCE group maintained a lower peripheral fatigue status.

A previous study demonstrated that an electrokinetically modified water (EMW) beverage can improve skeletal muscle function [34]. The source of bioactivity in EMW is thought to involve the presence of charge-stabilized nanostructures (CSNs), which by virtue of their size, charge, and stability affect constituents of the cell membrane [35, 36]. During exercise, increased lactate levels in exercising muscle and blood may trigger sensations of pain and discomfort, thus elevating perceptions of exertion [20, 37]. Ammonia is fatigue factor, whereby reduction of blood ammonia could prevent muscle catabolism and muscle damage [20]. Glucose is the main energy source during exercise and the glucose amount in the serum, liver, or muscle can greatly affect the body's exercise fatigue resistance [38]. In the current study, NCE is able to reduce lactate and ammonia accumulation while improving glucose utilization. This implies that NCE supplementation can ameliorate exercise fatigue and promote recovery.

Although studies on the effect of curcumin in reducing muscle damage are common, there is a lack of study reflecting evidence of its effect on exercise performance. One recent study showed that curcumin supplementation for 6 weeks in Wistar rats on a treadmill training model is able to affect exercise performance by regulating the NF-*κ*B and Nrf2 pathways [39]. In our study, we found that the PVGRF in the NCE group was lower than the placebo group after 4 weeks of supplementation. This finding could imply that the NCE group in DJH 70% drop height lightly loaded the lower extremities. NCE groups could reduce peak vertical ground reaction force at drop jumps. This implies that NCE may have the potential to reduce the risk of musculoskeletal system injury at this height (DJH 70%) in drop jumps. NCE supplementation also increases the ground contact time at DJH 100%, suggesting that the lower body muscle strength increased after 4-week treatment. These findings are consistent with our previous animal study showing that curcumin supplementation reduced fatigue after exercise [6]. However, this approach carries limitations and cannot show direct causative relationships between NCE supplementation effects on whole exercise system or systemic variables. Nevertheless, future clinical studies on programmed exercise prescriptions in combination with NCE supplementation would be important.

## 5. Conclusions

Curcumin is an important nutrient supplement with applications in reducing muscle damage associated with exercise training. There is much interest in generating nanobubbles, which can enable new uses of ultrasound contrast agents in molecular imaging and drug delivery, particularly for cancer applications [40]. To our knowledge, our study is the first using NBs on curcumin extraction. In the current study, we find that NCE can also play a role in physiological protection from the risk of musculoskeletal system injury due to explosiveness exercise. Fitness training has become popular in recent years because people recognize the positive effects of aerobic and endurance exercise on health promotion and physiological maintenance. Our data shows that NCE is beneficial for the reduction of fatigue and musculoskeletal system injury. Taken together, our results suggest that NCE, high quality form of curcumin, may be a potential nutrient supplement option to be used with aerobic and anaerobic exercises.

## Figures and Tables

**Table 1:** Effect of supplementation on the biochemical serum profile levels during posttest assessment.

Parameter	Placebo	NCE	*p*-value
AST (U/L)	16.4 ± 0.4	15.6 ± 0.3	0.1590
ALT (U/L)	9.5 ± 0.3	6.1 ± 0.5*∗*	0.0002
ALP (U/L)	89.4 ± 5.3	63.8 ± 2.3*∗*	0.0013
LDH (U/L)	176.4 ± 4.07	176.4 ± 4.01	0.9991
CK (U/L)	73.6 ± 6.5	63.7 ± 3.6	0.2136
Albumin (g/L)	45.6 ± 0.2	45.7 ± 0.3	0.6469
TP (g/L)	74.5 ± 0.8	74.9 ± 0.8	0.7461
BUN (mmol/L)	13.1 ± 0.2	12.4 ± 0.4	0.1253
UA (mmol/L)	4.8 ± 0.1	4.9 ± 0.1	0.3051
Glucose (mg/dL)	77.6 ± 1.8	78.4 ± 1.8	0.7744
TC (mg/dL)	164± 6	153 ± 7	0.2411
TG (mg/dL)	82 ± 4	56 ± 4*∗*	0.0006
HDL (mg/dL)	48 ± 1	57 ± 2*∗*	0.0017
LDL (mg/dL)	105 ± 10	86 ± 6	0.1450

Data are mean ± SEM and n = 6 subjects/group. The asterisk (*∗*) in the same row indicates a significant difference at p<0.05 by One-Way ANOVA. AST: aspartate aminotransferase; ALT: alanine aminotransferase; ALP: alkaline phosphatase; LDH: lactic dehydrogenase; CK: creatine kinase; TP: total protein; BUN: blood urea nitrogen; UA: uric acid; TC: total cholesterol; TG: triglycerides; HDL: high-density lipoprotein; LDL: low-density lipoprotein

**Table 2:** General characteristics of the body composition.

Characteristic	Treatment	Pre-test	Post-test	*p* values		
				Main effect (Sup)	Main effect (Times)	Interaction (Sup x Times)
Weight (kg)	Placebo	57.1 ± 5.1	56.8 ± 5.2	0.8554	0.9262	0.9785
	NCE	56.5 ± 7.6	56.4 ± 8.5			

BMI (kg/m^2^)	Placebo	20.7 ± 2.5	20.6 ± 2.5	0.9602	0.9602	0.9829
	NCE	20.8 ± 2.9	56.7 ± 3.3			

BMR (kcal/day)	Placebo	1265 ± 121	1261 ± 97	0.5452	0.9304	0.9944
	NCE	1237 ± 112	1232 ± 129			

FFM (kg)	Placebo	38.8 ± 3.6	38.5 ± 3.0	0.3989	0.8706	0.9567
	NCE	37.6 ± 3.2	37.4 ± 3.6			

Fat Mass (%)	Placebo	26.7 ± 2.6	26.5 ± 2.8	0.6386	0.9374	0.9922
	NCE	27.5 ± 5.2	27.4 ± 5.1			

Water content (%)	Placebo	50.2 ± 0.9	50.1 ± 0.9	0.5213	0.9067	0.9533
	NCE	49.8 ± 1.8	49.7 ± 1.7			

Bone salts (kg)	Placebo	2.98 ± 0.53	3.07 ± 0.33	0.4771	0.8116	0.8116
	NCE	3.15 ± 0.46	3.15 ± 0.46			

The main effect of supplementation (Sup) refers to the NCE and placebo treatment and the main effect of time refers to the two-time point assessments (pre-test, post-test). Data are presented as the mean ± SEM for *n*=6 in each group. A two-way ANOVA was used and a *p* value <0.05 was considered significantly different. The *post-hoc* test was performed by a repeated Student's *t*-test between groups at the same time point and significance (*∗*) was set to an alpha level of 0.05.

**Table 3:** Effect of NCE supplementation on drop jumps (DJs) with kinetic and kinematic data.

Characteristic	Treatment	Pre-test	Post-test	*p* values		
				Main effect (Sup)	Main effect (Times)	Interaction (Sup x Times)
Jump High (cm) DJH 70%	Placebo	18.6 ± 1.46	19.0 ± 0.62	0.2571	0.1613	0.2342
	NCE	18.6 ± 1.97	22.7 ± 1.72			

Jump High (cm) DJH 100%	Placebo	19.3 ± 0.92	19.3 ±0.84	0.4860	0.4095	0.4068
	NCE	19.1 ± 1.73	21.5 ± 1.94			

Jump High (cm) DJH 130%	Placebo	17.7 ± 1.11	18.1 ± 0.8	0.1191	0.3242	0.4871
	NCE	19.0 ± 1.83	21.5 ± 1.68			

RSI (cm/s) DJH 70%	Placebo	49.0 ± 3.27	44.8 ± 5.15	0.0895	0.2911	0.8004
	NCE	41.2 ± 6.51	34.4 ± 4.87			

RSI (cm/s) DJH 100%	Placebo	51.1 ± 2.53	43.8 ± 5.67	0.1604	0.5553	0.3527
	NCE	39.8 ± 5.7	41.4 ± 4.23			

RSI (cm/s) DJH 130%	Placebo	47.0 ± 3.59	40.1 ± 4.74	0.4327	0.5115	0.3488
	NCE	39.5 ± 4.35	40.8 ± 4.2			

Contact Time (ms) DJH 70%	Placebo	0.38 ± 0.02	0.45 ± 0.05	0.0799	0.1283	0.3224
	NCE	0.48 ± 0.05	0.80 ± 0.23			

Contact Time (ms) DJH 100%	Placebo	0.38 ± 0.03	0.47 ± 0.05	0.0379	0.1238	0.4630
	NCE	0.50 ± 0.03	0.53 ± 0.03 *∗*			

Contact Time (ms) DJH 130%	Placebo	0.39 ± 0.04	0.48 ± 0.05	0.0600	0.1112	0.5939
	NCE	0.49 ± 0.04	0.54 ± 0.03			

PVGRF (BW) DJH 70%	Placebo	3.1 ± 0.51	2.6 ± 0.29	0.2849	0.1383	0.8394
	NCE	2.8 ± 0.33	2.2 ± 0.07 *∗*			

PVGRF (BW) DJH 100%	Placebo	3.7 ± 0.95	2.7 ± 0.29	0.5291	0.1338	0.6413
	NCE	3.1 ± 0.35	2.6 ± 0.2			

PVGRF (BW) DJH 130%	Placebo	4.2 ± 0.65	3.2 ± 0.22	0.4605	0.0662	0.6376
	NCE	3.7 ± 0.42	3.1 ± 0.28			

(1) The posttest was assessed after 4 weeks of supplementation.

(2) The main effect of supplementation (sup) refers to the NCE and placebo treatment and the main effect of time is the distance recorded at 2 points (pre- and post-). Data are presented as the mean ± SEM for *n*=6 in each group. Statistical analysis was using a Two-Way ANOVA. A *p* value <0.05 was considered significantly different. The *post-hoc* test was performed by repeated Student's *t*-test between groups at the same time point and significance (*∗*) set at an alpha level of 0.05. Drop jumps high (DJH); reactive strength index (RSI); peak vertical ground reaction force (PVGRF).

## Data Availability

The data used to support the findings of this study are included within the article.
